# A JROD survey: nationwide overview of radiotherapy data from 2015 to 2021

**DOI:** 10.1093/jrr/rrad067

**Published:** 2023-09-20

**Authors:** Hisateru Ohba, Yoshihiro Nakada, Hodaka Numasaki, Kensuke Umehara, Junko Ota, Yasuo Okuda, Teruki Teshima, Kazuhiko Ogawa, Katsumasa Nakamura, Japanese Society for Radiation Oncology Database Committee

**Affiliations:** National Institutes for Quantum Science and Technology, 4-9-1 Anagawa, Inage-ku, Chiba-shi, Chiba 263-8555, Japan; National Institutes for Quantum Science and Technology, 4-9-1 Anagawa, Inage-ku, Chiba-shi, Chiba 263-8555, Japan; Department of Medical Physics and Engineering, Osaka University Graduate School of Medicine, 1-7 Yamadaoka, Suita, Osaka 565-0871, Japan; National Institutes for Quantum Science and Technology, 4-9-1 Anagawa, Inage-ku, Chiba-shi, Chiba 263-8555, Japan; Department of Medical Physics and Engineering, Osaka University Graduate School of Medicine, 1-7 Yamadaoka, Suita, Osaka 565-0871, Japan; National Institutes for Quantum Science and Technology, 4-9-1 Anagawa, Inage-ku, Chiba-shi, Chiba 263-8555, Japan; Department of Medical Physics and Engineering, Osaka University Graduate School of Medicine, 1-7 Yamadaoka, Suita, Osaka 565-0871, Japan; National Institutes for Quantum Science and Technology, 4-9-1 Anagawa, Inage-ku, Chiba-shi, Chiba 263-8555, Japan; Osaka Heavy Ion Therapy Center, 3-1-10 Otemae, Chuo-ku, Osaka 540-0008, Japan; Department of Radiation Oncology, Osaka University Graduate School of Medicine, 2-2 Yamadaoka, Suita, Osaka 565-0871, Japan; Department of Radiation Oncology, Hamamatsu University School of Medicine, 1-20-1 Handayama, Higashi-ku, Hamamatsu, Shizuoka 431-3192, Japan

**Keywords:** JROD, registry survey, radiotherapy, radiotherapy institution

## Abstract

The purpose of this survey was to examine the status of radiotherapy in Japan based on the cases registered in the Japanese Radiation Oncology Database (JROD), from 2015 to 2021, and to provide basic data to help improve the usefulness of the JROD in the future. The study population consisted of patients who underwent radiotherapy between 2014 and 2020 and did not opt out of the study. The survey item data analyzed in this study were entered into the database at each radiotherapy institution by referring to medical records from the preceding year. Our results show that the number of registered radiotherapy institutions and cases increased by ~50% in 2019 compared to those in 2015 (to 113 institutions and 60 575 cases, respectively). Among the survey item categories, the registration rate was lowest for prognostic information (13.9% on average over the 7-year period). In terms of the Japanese Society for Radiation Oncology disease site, the breast; lung, trachea and mediastinum and urogenital sites accounted for >50% of the total cases. The average survival and mortality rates over the 7-year study period were 67.4 and 17.4%, respectively. The X-ray radiotherapy completion rate exceeded 90% for all years and across all disease categories. ^192^Ir-based brachytherapy and ^223^Ra-based radionuclide therapy accounted for an average of 61.9 and 44.6%, respectively, of all corresponding cases over the 7-year period. In conclusion, this survey enables us to infer the actual status of radiotherapy in Japan based on the analysis of relevant nationwide data.

## INTRODUCTION

In December 2013, the Cancer Registration Promotion Act was enacted in Japan, which includes provisions for national cancer registration, the utilization and provision of relevant cancer-related information and the protection of such information. Additionally, it also established regulations for promoting hospital cancer registration, which have been in effect since January 2016 [1]. This law made it mandatory for radiotherapy institutions to report data on patients diagnosed with cancer to the prefectural governor, leading to the establishment of the National Cancer Registry Database in the National Cancer Center.

Furthermore, the Japan Surgical Society, in collaboration with various other surgical societies, established the National Clinical Database (NCD) in 2010 with the aim of achieving a better understanding of the current state of surgical care in Japan. In addition to information on surgeries, related treatments and autopsies in general, this database has also been collecting information on gastric, esophageal and lung cancers since 2011 [2].

Based on these clinical registries, the Japanese Society for Radiation Oncology (JASTRO) established the Japanese Radiation Oncology Database (JROD) in 2015 as part of a research project to understand the current status of radiotherapy interventions and to improve the quality of radiotherapy provided to patients. The database is managed by our institution (QST), which was also commissioned by the JASTRO [3].

The purpose of this survey was to clarify the status of radiotherapy in Japan between 2015 and 2021 and to provide basic data to help improve the usefulness of the JROD.

## MATERIALS AND METHODS

The survey included data from 2015 to 2021 obtained from 846 radiotherapy institutions that perform radiotherapy in Japan as determined based on the Ministry of Health, Labour and Welfare’s surveys of medical institutions conducted in 2014, 2017 and 2020. The study population consisted of patients who underwent radiotherapy between 2014 and 2020 and chose not to opt out of the study. Each radiotherapy institution registered the relevant data regarding the survey items based on medical records from January to December of the preceding year using a questionnaire format through a dedicated registration website. The four major categories of survey items were as follows:

A. Patient information (nine items: gender, age at initiation of radiation therapy, multiple cancer incidence, treatment history, etc.)

B. Disease information (28 items: disease name, primary site, stage classification, TNM classification, JASTRO disease site, etc.)

C. Treatment information (49 items: treatment strategy, total radiation dose, fractionation, treatment management fee, treatment completion status, etc.)

D. Prognostic information (25 items: survival/death status, recurrence, adverse events, second primary cancer incidence, etc.)

The following aspects were analyzed in this study: (i) the number and proportion of registered radiotherapy institutions and cases by prefecture; (ii) registration rates for each survey item; (iii) number and proportion of patients by disease category; (iv) survival status (The number of survival status represents the last confirmed survival or dead status between the date of irradiation and the date of case registration.); (v) X-ray radiotherapy completion rate and (vi) brachytherapy and radionuclide therapy registration rates. This study was conducted as a collaborative research project between QST and JASTRO, and it was approved by the institutional review board of the QST (approval number: 15-014).

## RESULTS

Table 1 presents the number of registered radiotherapy institutions and cases by prefecture. The number of registered radiotherapy institutions increased by 48.7% between 2015 and 2019 (from 76 in 2015 to a maximum of 113 in 2019). Similarly, the number of registered cases also increased by 49.0% (from 40 664 in 2015 to a maximum of 60 575 in 2019). However, only ~13% of the total number of radiotherapy institutions nationwide were registered. Moreover, there were no registered radiotherapy institutions in six prefectures: Shimane, Hiroshima, Kagawa, Kochi, Oita and Miyazaki.

**Table 1:** Number of radiotherapy institutions and cases registered in the JROD

Prefecture	MHLW			JROD													
	2020^**a**^	2017^**a**^	2014^a^	2021		2020		2019		2018		2017		2016		2015	
	Institutions^**b**^	Institutions^**b**^	Institutions^**b**^	Institutions	Cases	Institutions	Cases	Institutions	Cases	Institutions	Cases	Institutions	Cases	Institutions	Cases	Institutions	Cases
Hokkaido	38	40	37	5	2395	4	2263	4	2286	4	1960	3	874	3	987	2	849
Aomori	13	12	14	1	674	1	643	1	659	1	681	1	685	1	685	1	573
Iwate	11	11	12	3	1558	3	1158	3	1347	3	1494	3	1513	3	1577	2	1248
Miyagi	14	14	14	1	572	1	750	1	243	1	310	1	835	0	0	0	0
Akita	11	11	11	1	165	1	130	1	148	1	171	1	158	1	137	0	0
Yamagata	6	7	7	1	643	1	709	1	710	1	700	1	684	1	595	1	583
Fukushima	14	12	13	1	632	1	639	1	677	1	851	1	794	1	511	1	548
Ibaraki	17	20	21	2	871	2	931	2	897	3	1425	0	0	1	755	0	0
Tochigi	10	10	10	1	779	1	497	2	1031	1	489	1	536	0	0	0	0
Gunma	12	13	14	2	1047	2	997	2	1149	2	1053	2	995	2	1522	0	0
Saitama	26	26	24	4	1865	4	1957	4	1849	4	1642	4	1304	2	754	2	689
Chiba	32	33	34	6	2421	4	2262	3	1999	4	2359	4	2326	5	2289	2	357
Tokyo	78	82	78	13	9057	13	9171	14	10 690	12	10 068	12	8593	13	8832	12	8516
Kanagawa	43	43	40	6	3808	6	3773	6	3937	6	4379	6	3502	6	4340	4	2739
Niigata	17	16	17	3	748	1	637	3	733	3	863	3	797	3	664	1	296
Toyama	11	11	11	0	0	3	757	0	0	0	0	0	0	0	0	0	0
Ishikawa	10	10	10	0	0	0	0	1	207	1	222	0	0	1	241	0	0
Fukui	7	7	7	1	366	1	298	1	226	1	256	1	286	1	402	1	2
Yamanashi	5	5	5	0	0	0	0	1	665	2	813	1	859	1	158	1	188
Nagano	13	14	16	2	737	2	695	2	689	2	756	1	594	3	1090	2	795
Gifu	14	15	14	1	551	1	611	1	568	1	566	1	512	1	643	2	1039
Shizuoka	28	28	28	5	3493	4	2730	4	2637	3	2239	3	599	4	2662	4	2612
Aichi	44	44	43	7	3131	3	1575	6	2518	6	2561	6	2758	6	2432	6	2345
Mie	12	11	13	2	1080	2	1054	2	977	2	904	2	758	2	447	2	817
Shiga	12	12	11	2	906	2	1022	2	1066	2	1128	2	971	3	1340	3	1318
Kyoto	17	18	17	4	2378	4	2297	4	2198	3	959	3	896	3	870	1	364
Osaka	67	65	64	8	5974	7	5219	9	5746	7	5536	8	4968	6	3411	5	2962
Hyogo	33	37	36	4	1970	5	2410	4	2138	4	3223	2	2151	6	4591	5	4554
Nara	7	7	9	2	1164	2	1179	2	931	2	899	2	1120	2	1150	2	1107
Wakayama	10	10	10	2	656	2	678	2	718	1	122	2	662	1	146	1	414
Tottori	7	7	7	1	429	2	636	1	371	2	549	2	579	1	156	0	0
Shimane	6	6	6	0	0	0	0	0	0	0	0	0	0	0	0	0	0
Okayama	12	13	13	3	1666	3	1597	4	1733	4	1782	4	1976	3	1656	3	1609
Hiroshima	19	21	19	0	0	0	0	0	0	0	0	0	0	0	0	0	0
Yamaguchi	13	14	13	1	455	1	459	1	431	0	0	0	0	0	0	0	0
Tokushima	6	6	6	0	0	1	861	1	861	1	833	1	703	1	556	1	631
Kagawa	8	9	9	0	0	0	0	0	0	0	0	0	0	0	0	0	0
Ehime	11	12	12	1	432	1	359	1	402	1	630	1	613	2	870	1	449
Kochi	6	6	6	0	0	0	0	0	0	0	0	0	0	0	0	0	0
Fukuoka	34	33	32	7	3393	7	3583	9	4048	9	3999	8	3482	8	3430	5	1668
Saga	6	6	5	2	565	2	401	2	451	2	472	2	507	2	507	1	224
Nagasaki	10	9	9	0	0	1	675	1	623	1	651	1	617	1	627	1	702
Kumamoto	12	13	13	1	489	1	486	1	502	1	516	1	508	1	552	1	466
Oita	10	14	14	0	0	0	0	0	0	0	0	0	0	0	0	0	0
Miyazaki	8	7	8	0	0	0	0	0	0	0	0	0	0	0	0	0	0
Kagoshima	15	16	15	0	0	0	0	1	566	1	559	0	0	2	882	0	0
Okinawa	9	10	7	2	926	2	963	2	948	2	777	2	693	2	783	0	0
Total	824	846	834	108	57 996	104	57 062	113	60 575	108	59 397	99	50 408	105	53 250	76	40 664

^a^Data from the Survey of Medical Institutions carried out by the Ministry of Health, Labour and Welfare (MHLW)

^b^Number of hospitals and clinics providing external beam radiation therapy

Table 2 presents the rates of registration of data according to the study categories and items. The registration of patient information exceeded 50% in all survey years, while the registration of prognostic information had the lowest average rate of 13.9% of all four major categories. Nevertheless, the registration rate for the ‘Date last verified’ item in this category averaged 51.3% over the 7-year study period.

**Table 2:** Study category and item data registration ratios (%)

Category	2021	2020	2019	2018	2017	2016	2015	Average
A. Patient information (9 items)	56.9	57.0	55.5	55.1	54.9	52.4	50.3	54.6
Double cancer	62.9	63.3	56.9	50.4	49.6	40.9	34.5	51.2
B. Disease information (28 items)	32.9	33.5	32.7	32.4	31.5	32.1	28.1	31.9
Disease name	97.9	98.4	96.2	97.5	96.5	96.9	95.5	97.0
C. Treatment information (49 items)	33.1	33.6	31.3	30.9	29.6	27.0	26.7	30.3
Radiation therapy management fee (initial billing)	48.5	48.6	43.9	39.2	33.0	21.9	20.7	36.6
Radiation therapy management fee (second billing)	11.9	13.6	10.7	7.7	8.0	5.3	5.8	9.0
Radiation therapy completion rate	37.3	35.7	31.2	33.8	30.3	32.0	30.1	32.9
D. Prognosis information (25 items)	16.3	17.8	14.1	12.6	12.8	12.1	11.7	13.9
Mortality/survival status	47.9	64.6	20.1	18.6	22.8	21.6	19.6	30.7
Date last verified	73.9	79.2	55.1	45.6	42.7	31.6	31.1	51.3
Presence or absence of relapse	12.7	12.5	8.6	8.0	7.8	10.8	8.7	9.9
Presence or absence of adverse event	42.4	43.1	31.5	24.5	21.6	12.4	11.0	26.6

Table 3 shows the number and ratio of registered cases according to the JASTRO disease site. The three sites with consistently high registration rates across all survey years were: breast; lung, trachea and mediastinum and urogenital, accounting for >50% of all registered cases.

**Table 3:** JASTRO disease site data

JASTRO disease site	2021		2020		2019		2018		2017		2016		2015	
	*n*	Ratio (%)	*n*	Ratio (%)	*n*	Ratio (%)	*n*	Ratio (%)	*n*	Ratio (%)	*n*	Ratio (%)	*n*	Ratio (%)
Cerebrospinal	2218	3.8	2544	4.5	2715	4.5	2599	4.4	2025	4.0	2756	5.2	1954	4.8
Head and neck (including thyroid)	5692	9.8	5846	10.2	5977	9.9	5538	9.3	4735	9.4	5327	10.0	4154	10.2
Esophagus	2746	4.7	2932	5.1	3063	5.1	2959	5.0	2598	5.2	2870	5.4	2355	5.8
Lung, trachea and mediastinum	11 261	19.4	10 673	18.7	11 326	18.7	11 075	18.6	9697	19.2	9691	18.2	7471	18.4
Breast	11 752	20.3	11 561	20.3	12 247	20.2	12 702	21.4	11 334	22.5	10 473	19.7	7308	18.0
Liver, biliary tract and pancreas	2215	3.8	2293	4.0	2340	3.9	2411	4.1	1865	3.7	2119	4.0	1587	3.9
Gastric, small intestine and colorectal	2969	5.1	3027	5.3	3003	5.0	2905	4.9	2385	4.7	2428	4.6	1900	4.7
Urogenital (including prostate)	8329	14.4	7733	13.6	8838	14.6	8745	14.7	6578	13.0	7634	14.3	5662	13.9
Gynecologic	3565	6.1	3359	5.9	3467	5.7	3290	5.5	2884	5.7	3363	6.3	2854	7.0
Hematopoietic and lymphatic	3418	5.9	3292	5.8	3518	5.8	3247	5.5	2918	5.8	2985	5.6	2222	5.5
Skin, bone and soft tissue	2556	4.4	2501	4.4	2802	4.6	2698	4.5	2323	4.6	2590	4.9	2437	6.0
Other (malignant)	728	1.3	792	1.4	833	1.4	783	1.3	783	1.6	652	1.2	536	1.3
Benign	547	0.9	509	0.9	446	0.7	445	0.7	283	0.6	362	0.7	224	0.6
Total	57 996	100.0	57 062	100.0	60 575	100.0	59 397	100.0	50 408	100.0	53 250	100.0	40 664	100.0

Table 4 shows the survival status data for each survey year. The overall average survival ratio was 67.4%, comprising survival with tumor (19.9%), survival with unknown tumor (26.5%) and survival without tumor (21.0%). The average mortality ratio was 17.4%, including death due to the primary disease (including the primary tumor; 12.0%), death due to other diseases (0.8%) and unexplained death (4.5%).

**Table 4:** Survival status data.

Status^b^			2021		2020		2019		2018		2017		2016		
			*n*	Ratio^a^	*n*	Ratio^a^	*n*	Ratio^a^	*n*	Ratio^a^	*n*	Ratio^a^	*n*	Ratio^a^	
Survival with tumor			5955	23.6	4598	13.8	2898	23.9	1858	16.8	1514	13.2	2875	25.0	
Survival with unknown tumor			5203	20.6	13 484	40.5	2466	20.3	3205	29.0	3191	27.8	2225	19.3	
Survival without tumor			5319	21.0	5406	16.3	3053	25.1	1963	17.8	2564	22.4	3202	27.8	
Death due to the primary disease (including the primary tumor)			1310	5.2	1195	3.6	2161	17.8	1395	12.6	1339	11.7	1901	16.5	
Death due to other diseases			165	0.7	245	0.7	109	0.9	91	0.8	103	0.9	116	1.0	
Unexplained death			1059	4.2	773	2.3	210	1.7	714	6.5	744	6.5	423	3.7	
Patient lost to follow-up			6262	24.8	7564	22.7	1252	10.3	1817	16.5	2009	17.5	779	6.8	
Total			25 273	100.0	33 265	100.0	12 149	100.0	11 043	100.0	11 464	100.0	11 521	100.0	

Survival with tumor = the patient survives with tumor-bearing based on the diagnosis, Survival with unknown tumor = the patient survives, although it is uncertain whether the tumor remains, Survival without tumor = the patient survives without any signs or symptoms of tumor based on the diagnosis, Death due to the primary disease (including the primary tumor) = the patient died due to the target disease (including recurrence or metastasis), Death due to other diseases = the patient died due to other diseases, or the primary tumor was controlled, but death occurred due to secondary tumor, Unexplained death = the patient died with an unknown cause, Patient lost to follow-up = the patient’s status is unknown, whether they are alive or deceased.

^a^Ratio of each status number to total number.

^b^Definition of survival status.

Table 5 presents the X-ray radiotherapy completion rate data. Each year, the overall treatment completion rate, including cases with therapy cessation for >8 days, consistently exceeded 90%.

**Table 5:** X-ray radiotherapy completion rates (%)

	2021	2020	2019	2018	2017	2016	2015
Completion as planned	96.4	96.4	91.2	89.5	89.7	91.5	95.9
Completion (with therapy cessation of >8 days)	0.4	0.4	1.0	1.1	1.6	1.6	0.3
Canceled after completion of <50% of planned therapy	1.1	1.3	1.5	1.5	1.6	1.6	0.6
Canceled after completion of >50% of planned therapy	1.8	1.6	1.9	1.9	2.0	2.3	0.7
Canceled with degree of completion unknown	0.0	0.1	0.1	0.1	0.3	0.2	2.4
Other	0.1	0.2	0.1	0.0	0.0	0.1	0.0
Unknown	0.2	0.0	4.3	5.8	4.9	2.8	0.0

Table 6 shows the number of registered cases of brachytherapy and radionuclide therapy during the study period. With regard to brachytherapy, the 7-year average shows that ^192^Ir-based therapy accounted for 61.9% and ^125^I-based therapy accounted for 33.6% of the registered cases. With regard to radionuclide therapy, the 7-year average shows that ^223^Ra-based therapy accounted for 44.6% and ^131^I-based therapy accounted for 43.4% of registered cases.

**Table 6:** Ratios of registered cases of brachytherapy and radioisotope therapy (%)

	2021	2020	2019	2018	2017	2016	2015
Brachytherapy							
Ir-192	71.0	64.6	56.1	56.9	62.5	55.6	66.5
I-125	22.6	33.1	39.9	36.7	31.2	41.8	29.7
Co-60	3.1	1.5	3.5	4.3	5.8	2.5	3.0
Au-198	2.4	0.1	0.3	2.1	0.5	0.1	0.8
Sr-90	0.8	0.7	0.3				
Total number	1559	1832	1766	1688	1297	1582	1000
Radioisotope therapy							
I-131	50.4	22.8	39.7	24.6	67.0	64.1	34.7
Sr-89	0.4	4.8	5.8	4.0	8.8	23.3	12.2
Y-90	0.8	4.1	1.0	2.4	5.5	12.6	0.0
Ra-223	48.3	68.3	53.5	69.0	18.7	0.0	53.1
Total number	240	145	310	252	91	103	98

## DISCUSSION

The results of this JROD survey showed that the number of registered radiotherapy institutions exhibited an increasing trend until 2019 after which it has remained relatively stable (Table 1). The observed increase can be attributed to the widespread implementation of radiotherapy- and radiology-related information systems at various radiotherapy institutions, which have been made compatible with the data output format of the JROD. On the other hand, several factors can explain the reasons for the subsequent lack of growth in the number of registered radiotherapy institutions, including (i) the complexity of data entry, (ii) the requirements of an institutional review board, (iii) restrictions on connecting to external networks from within institutions and (iv) the perceived lack of benefits of registering the relevant data. To enhance the usefulness of the database in the future, it will be necessary to increase the number of registered institutions and cases by establishing mechanisms such as collaboration with a medical specialist system like the NCD, or making registration a requirement for JASTRO-certified institutions as part of a larger academic/social project. Furthermore, it would also be necessary to implement systems that benefit the registered institutions, such as a feedback system [2] that provides mechanisms for analyzing the accumulated data and returning the results to the clinical sites, as NCD does, in this database system.

The lower registration rate for prognostic information, as indicated in Table 2, can be attributed to the higher proportion of items that require free-text input. Additionally, regarding the follow-up registration initiated in 2020, it is likely that the specifications of the database cause the information registered in the previous year to be overwritten, making it difficult to track prognosis. This limitation may discourage radiotherapy institutions from inputting the data, thereby affecting the overall registration rate.

Notably, the disease-specific registration rates shown in Table 3 for the years 2016, 2018 and 2020 exhibited similar trends to those for the proportions of new cases reported in the JASTRO structural surveys (2015, 2017 and 2019) [4–6].

Tables 4 and 5 show that there were minimal changes in the survival status and the X-ray radiotherapy completion rate over time, confirming the effectiveness and stability of the radiation treatments provided. The status of survival was more than double the number of registrations in 2020 and 2021 compared to previous years, which can be attributed to the inclusion of survival as a mandatory item in the prognostic survey introduced in 2020 [7].

The registration rate for ^192^Ir-based brachytherapy in 2021 increased by 6.4% compared to that in 2020 (Table 6). This increase can be attributed to factors such as the revision of medical fee rates for high-dose rate iridium irradiation therapy, with an increase of 2000 medical fee points, as well as an increase of 900 medical fee points for image-guided brachytherapy, implemented in the April 2020 medical fee revision [8]. Regarding ^223^Ra-based radionuclide therapy, the registration rate increased significantly in 2018 (to 69.0%) compared to that in the previous year. This can be attributed to the introduction of a new management fee of 2630 points for ^223^Ra-based radionuclide therapy for castration-resistant prostate cancer with bone metastasis as part of the April 2018 medical fee revision [9].

In conclusion, this study analyzed the JROD data regarding various aspects of radiotherapy in Japan over the 7-year period from 2015 to 2021. Our results provide an overview of the state of radiotherapy in Japan in this period; however, considering that the current number of registered radiotherapy institutions accounted for only 13% of all such institutions in Japan, increasing the number of registered radiotherapy institutions and the registration rate for each survey item will be necessary for more detailed and accurate inferences regarding the relevant diseases and treatments.
